# Role of Insulin in Neurotrauma and Neurodegeneration: A Review

**DOI:** 10.3389/fnins.2020.547175

**Published:** 2020-09-23

**Authors:** Michael Shaughness, Deanna Acs, Fiona Brabazon, Nicole Hockenbury, Kimberly R. Byrnes

**Affiliations:** ^1^Neuroscience Program, Uniformed Services University of the Health Sciences, Bethesda, MD, United States; ^2^Department of Anatomy, Physiology and Genetics, Uniformed Services University of the Health Sciences, Bethesda, MD, United States

**Keywords:** Alzheimer’s disease, insulin, inflammation, microglia, neurons, Parkinson’s disease, spinal cord injury, traumatic brain injury

## Abstract

Insulin is a hormone typically associated with pancreatic release and blood sugar regulation. The brain was long thought to be “insulin-independent,” but research has shown that insulin receptors (IR) are expressed on neurons, microglia and astrocytes, among other cells. The effects of insulin on cells within the central nervous system are varied, and can include both metabolic and non-metabolic functions. Emerging data suggests that insulin can improve neuronal survival or recovery after trauma or during neurodegenerative diseases. Further, data suggests a strong anti-inflammatory component of insulin, which may also play a role in both neurotrauma and neurodegeneration. As a result, administration of exogenous insulin, either via systemic or intranasal routes, is an increasing area of focus in research in neurotrauma and neurodegenerative disorders. This review will explore the literature to date on the role of insulin in neurotrauma and neurodegeneration, with a focus on traumatic brain injury (TBI), spinal cord injury (SCI), Alzheimer’s disease (AD) and Parkinson’s disease (PD).

## Introduction

Insulin is a large hormone (5,808 Da) typically produced by the pancreas and its passage into the brain is tightly regulated by saturable insulin transporters on the blood brain barrier (BBB). Insulin receptors (IR) are expressed by neurons and glia and mediate insulin signaling throughout the brain (Wozniak et al., 1993; Uemura and Greenlee, 2006; Chiu et al., 2008; Song et al., 2015). While it has been shown that select neurons can produce insulin *de novo* (Schechter et al., 1990; Saatman et al., 2008), the majority of insulin in the brain is from the blood. However, the ability of neurons to synthesize insulin suggests a necessary role of insulin in normal function and development.

Insulin signaling plays a role in global brain glucose metabolism (Bingham et al., 2002) and cerebral functions such as memory and cognition (Schulingkamp et al., 2000). The cognitive enhancing effects of insulin in people were first described in studies using systemic infusions of insulin under euglycemic hyperinsulinemic conditions (Kern et al., 2001). Infusions were given for a total of 360 min, during which subjects underwent memory (word recall) and selective attention tasks (Stroop test), with mood and bodily symptoms assessed by self-report. Subjects showed significantly enhanced memory performance, as measured by recalling more words from an orally presented list after a 1-min delay. Insulin improved performance on the Stroop interference task and this coincided with subjective reports of feelings to have less “difficulty in thinking.” The results suggested that insulin improved attention and working memory in healthy humans. This study carefully controlled for the administration effect of IV insulin, which alters blood glucose levels and contributes to negative metabolic states and hypoglycemia in certain patient populations.

In order to achieve the positive cognitive and mood effects of insulin, without altering systemic blood glucose levels, alternative delivery methods have been explored. Evaluation of memory in rodent models demonstrated elevated hippocampal neurogenesis and BDNF production following direct infusion of insulin to the cerebral ventricles (Haas et al., 2016). Intranasally delivered insulin significantly improved word-recall memory scores, mood assessments and self-confidence in healthy human subjects in 2004 study (Benedict et al., 2004). This demonstrated, for the first time, the beneficial properties of intranasal insulin without altering blood-glucose levels and weight. Further research has shown that insulin administration before bed, and presumably during memory consolidation, improved word recall (Ritze et al., 2018).

Intranasal delivery of insulin allows insulin to bypass the saturable BBB insulin delivery system and reach the brain directly via the olfactory and trigeminal nerve pathways and distribution into the cerebrospinal fluid (CSF) (Thorne et al., 1995). Thus, intranasal, and other direct CNS infusion methods, avoid the potential complication of insulin induced hypoglycemia seen with intravenous (IV) infusion. Insulin delivered intranasally comes into direct contact first with the olfactory sensory neurons dendritic processes, which are present in the upper nasal passage, and their axons, which are present in the spaces of the cribriform plate (Thorne et al., 1995; Thorne and Frey, 2001). Free nerve endings of branches from the trigeminal nerve are also present in the nasal epithelium (Finger et al., 1990). Insulin is transported along the olfactory and trigeminal nerves by intracellular pathways, via endocytosis by the nerve then anterograde transport, or extracellular pathways, via paracellular diffusion (Thorne et al., 1995; Baker and Spencer, 1986; Born et al., 2002; Renner et al., 2012; Lochhead et al., 2015; Lochhead et al., 2019).

Reduced sensitivity or resistance to insulin actions, via downregulation or loss of IR or reduced activity of insulin signaling pathway, contributes to worsened outcome in several neurological conditions, further highlighting the importance of insulin in the CNS. Insulin resistance is observed in various instances of neurotrauma (Karelina and Weil, 2016; Franklin et al., 2019; Kim et al., 2019) and neurodegenerative diseases (Diehl et al., 2017; de la Monte, 2012).

The focus of this review is therefore to characterize the current literature on the role of insulin in CNS disorders, with a focus on traumatic brain injury (TBI), spinal cord injury (SCI), and neurodegenerative diseases, specifically Alzheimer’s disease (AD) and Parkinson’s disease (PD).

## Insulin at the Cellular Level

### Neurons

**FIGURE 1:**
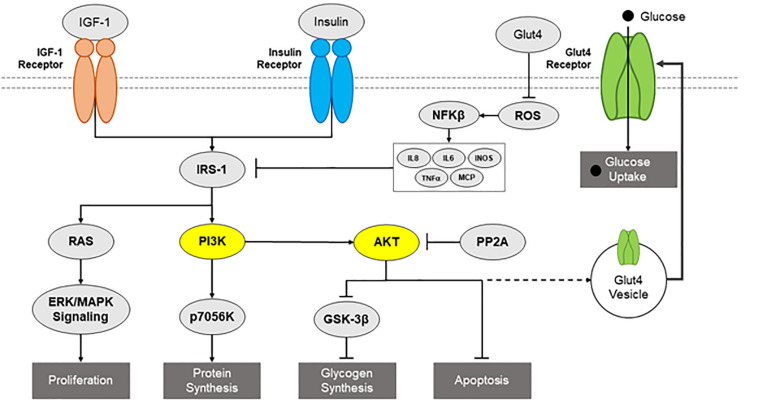
Diagram of insulin signaling pathways. Potential insulin signaling pathways, such as IGF-1 receptor, IR, IRS1, RAS, PI3K/AKT pathways, are presented, including relevant downstream consequences and inflammatory inhibitors.

Neurons interact with insulin through insulin, insulin-like growth factor 1 (IGF-1), and insulin/IGF-1 hybrid receptors. Insulin signaling is modulated through the tyrosine phosphorylation of cellular substrates, including several IR substrates (IRS) (Brummer et al., 2010), as well as other scaffold proteins (Taguchi and White, 2008), which initiate divergent signal transduction pathways (Saltiel and Pessin, 2002) (Figure 1). Insulin increases neuronal glucose uptake by increasing the translocation of GLUT 3 and 4 from the cytosol to the membrane (Grillo et al., 2009). Recently, studies have highlighted the interaction of insulin and neuronal GLUT4. GLUT4 is primarily expressed on neurons, particularly on those that are involved during periods of high energy demand, such as those found in the hippocampus (El Messari et al., 2002; McEwen and Reagan, 2004). Primary hippocampal neurons treated with 100 nM of insulin showed a significant increase in glucose utilization and this effect was blocked with a 100 μM dose of indinivar, a GLUT4 inhibitor, and indinivar alone had no effect on glucose utilization (Pearson-Leary et al., 2018). *In vivo* studies demonstrated insulin’s cognitive effects by showing that inhibition of endogenous intrahippocampal insulin impaired spatial working memory in the spontaneous alternation behavior in a 4-arm plus-maze (SA) and acute administration of intrahippocampal insulin enhances SA performance (McNay et al., 2010). To elucidate the involvement of hippocampal GLUT4 in insulin’s cognitive enhancing effects *in vivo* intrahippocampal administration of a selective inhibitor of GLUT4, indinavir sulfate, was co-administered with insulin 10 min prior to the SA task. The selective blocking of GLUT4 prevented insulin’s cognitive enhancing effects by returning performance on SA to that of controls, demonstrating the relationship between insulin and GLUT4 in improving cognition (Pearson-Leary et al., 2018). Additionally, insulin essentially acts as a neurotrophic factor as it has been repeatedly shown to support neuronal survival in a manner separate from its metabolic influence (Schechter et al., 1990; Tanaka et al., 1995; van der Heide et al., 2006). Insulin promotes neurite growth and formation by promoting α and β tubulin production, suggesting a crucial role in neurodevelopment and maintenance (van der Heide et al., 2006; Mergenthaler et al., 2013).

Insulin signaling is fundamental for proper functioning of neurons and memory formation and storage. There is an increase in IRs in synapses of the hippocampus following a short-term memory task (Zhao et al., 1999). Insulin also can regulate the endocytosis of AMPA receptors, thus playing a role in synaptic function and long-term depression (Man et al., 2000; Huang et al., 2004).

Primary rat cortical neurons have been used to mimic insulin resistance (Kim et al., 2011). Cortical neurons chronically treated with 20 nM showed a reduction in phosphorylation of AKT, p70S6K, and glycogen synthase kinase 3 beta (GSK-3β), but no significant change in ERK. Furthermore, when an upstream regulator of AKT, phosphatidylinositol 3-kinase (PI3K), was blocked during insulin pre-treatment, phosphorylation of AKT, p70S6K, and GSK-3β was restored, suggesting that resistance is mediated by alteration of PI3K signaling. These observations were confirmed in an *ex vivo* mouse model of diabetes, where cortical slices from diabetic BKS-db/db mice demonstrated no change in phosphorylation of AKT when treated with insulin. In contrast, cortical slices from controls did show a dose-dependent increase in AKT phosphorylation. These results indicate that chronic insulin stimulation results in decreased acute insulin-stimulated AKT activation; this is a situation that should be kept in mind when designing long-term therapeutic strategies utilizing insulin.

Recently, a novel non-insulin mediated driver of insulin resistance was proposed using an *in vitro* model of excitotoxicity (Pomytkin et al., 2019). Primary rat cortical neurons exposed to 100 μM of glutamate for 30 min demonstrated a rapid increase in calcium, nearly 2.5 fold increase above baseline, coinciding with a significant mitochondrial depolarization that reached a 1.6 fold decrease below baseline. These changes led to significantly decreased activation of the insulin signaling pathway, as measured by a significantly lower ratio of phosphorylated AKT to total AKT when compared to control cells, similar to that observed in insulin resistance. To investigate the effect glutamate excitotoxicity has on insulin signaling, cells were treated with 100 μM of insulin for 15 min after being stimulated with glutamate for 30 min. Pre-treatment with glutamate led to a significant reduction in the ratio of phosphorylated to total protein in IR β-subunit, AKT, mTOR, and GSK-3β compared to insulin treated only cells. These results suggest glutamate induced excitotoxicity acutely inhibits insulin mediated activation of the IR/AKT/mTOR pathway, leading to insulin resistance during periods of mitochondrial depolarization caused by glutamate-evoked massive influxes of calcium. These findings suggest that trauma and other situations in which cells experience excitotoxicity may also be periods of induced insulin resistance.

However, insulin may also be able to prevent injury and damage. Exposure of neuronal precursors to hypoxia induces cell death. This effect was blocked by the administration of insulin, in a PI3K/AKT pathway dependent manner (Zhao et al., 2007). Also, oxidative stress is a shared pathophysiological process implicated in TBI, SCI, and neurodegenerative disease, with lipid and protein oxidation altering the conformation and structure of membrane proteins, including GLUT3, thus decreasing neuronal glucose uptake and intracellular ATP (Butterfield et al., 2002). Using primary rat cortical neurons, researchers demonstrated that pretreatment with insulin was able to prevent oxidative stress-induced impairment of glucose accumulation and metabolism, which was inflicted by the addition of ascorbic acid and FeSO_4_ (Duarte et al., 2006). Further, using the glutamate excitotoxicity model described above, cortical neurons pretreated with 100 nM of insulin for 5 min before glutamate stimulation showed a reversal of glutamate induced increases in mitochondrial membrane depolarization, increased intracellular calcium and decreased the number of cells displaying damage (Krasil’nikova et al., 2019).

Neuronal injury and the ensuing adverse consequences on neurocognitive performance are important aspects of HIV/AIDS neuropathogenesis. The pathological protein implicated in HIV/AIDs is HIV-1 Vpr, and leads to neuronal death, as measured by loss of MAP-2-immunolabeled cell processes. Insulin was shown to have protective effects against HIV-Vpr-induced neurotoxicity in primary human neurons (Mamik et al., 2016). This was demonstrated by pre-treating human fetal neurons with varying concentrations of insulin for one hour before challenging with the neurotoxic protein HIV-1 Vpr. Forty-eight hours later, neurons treated with insulin displayed significantly more β-III tubulin, MAP2, and DAPI staining compared to HIV-Vpr treated only cells. These results suggest that, in the context of viral-mediated neurodegeneration, insulin acts as neurotrophic factor improving survival.

These studies demonstrate that the effects of insulin fall along a spectrum. On one end, chronic exposure to insulin can lead to insulin resistance and dysfunctional insulin signaling which are associated with the suboptimal post-insult recovery and the development of certain proteinopathies. On the other end, insulin shows promise as a preventative molecular therapeutic to ameliorate adverse outcomes associated with oxidative stress and viral-mediated neuronal pathophysiology. Additionally, studies have shown insulin to be neuroprotective. While these studies provide important insight into the potential therapeutic value of targeting the insulin pathway, the limitations of *in vitro* models need to be highlighted. Specifically, the *in vitro* cellular environment is much different than complex and heterogeneous *in vivo* settings. The doses administered *in vitro* may not be physiologically relevant in an animal model or human. Future work is needed to better explore the balance between therapeutic doses and those causing insulin resistance and to better investigate the mechanisms behind resistance, to better design treatment strategies.

### Astrocytes

The role of insulin in glial populations is significantly less studied than the neuron-insulin relationship. This may be attributed to the significantly higher expression of IR on neurons than on glia (Frolich et al., 1998). Nevertheless, an understanding of glial activity in response to insulin is crucial. Astrocytes are glial cells that outnumber neurons in the brain and play a crucial role in neural transmission by clearing glutamate from the synaptic cleft. After an injury, they adopt an activated phenotype that produces reactive oxygen species (ROS), excess cytokines, and forms the glial scar in more severe injuries (Myer et al., 2006).

The relationship between insulin and astrocytes has predominantly been studied in the context of metabolism and appetitive behavior (Brown and Ransom, 2007). Insulin promotes glycogen storage in astrocytes (Heni et al., 2011). Neurons lack glycogen stores, so this characteristic of astrocytes lends itself to a cooperative metabolic relationship between astrocytes and neurons (for review: Falkowska et al., 2015).

Cultured human astrocytes express functional insulin/IGF1 signaling (IIS) pathways. Full activation of the IIS pathway in human cultured astrocytes was identified using western blot analysis for expression of IRβ, IGF1Rβ, IRS1, IRS2, pAKT, Total AKT, p44/42, and MAPK following insulin treatment (1 μM recombinant human insulin) (Garwood et al., 2015).

Astrocytes have been shown *in vitro* to respond to insulin at low doses (1 nM) with increased production of IL-6 and IL-8, pro-inflammatory cytokines. However, this effect dissipates at higher doses (100 nM), suggesting that insulin plays a role in astrocyte inflammatory response as well as glycogen storage (Spielman et al., 2015). Low doses of insulin significantly reduced lipopolysaccharide (LPS)-induced inducible nitric oxide synthase (iNOS) expression and activation of NFκB in astrocytes (Li et al., 2013). An *in vitro* model of PD, using rat glioblastoma cell line C6, showed that one hour pre-treatment with insulin (100 nM) protects against 24 h MPP + (500 μM) stimulation-induced toxicity, as demonstrated by significantly decreased LDH and nitric oxide (NO) media release and iNOS and COX2 expression when compared to MPP + stimulated only cells (Ramalingam et al., 2017). Insulin also increased viabilty, measured by MTT assay, in primary human derived astrocytes exposed to a 24 serum starvation (1% fetal bovine serum in media) at 48 and 72 h (Shahriyary et al., 2018).

While currently understudied, insulin activity within astrocytes is clearly a potential mechanism of beneficial therapeutic effects. Astrocytes are the most abundant cells in the brain and provide a myriad of functions including maintenance of BBB integrity and the provision of metabolic support to neurons. Insulin’s therapeutic effect on astrocyte viability, physiology, and function in the context of neurological disorder and neurodegeneration is starting to garner interest and represents a novel avenue for theraputic intervention.

### Microglia

Microglia are the resident macrophage of the CNS and make up about 10–15% of the cells of the CNS. While similar in several aspects, microglia have different developmental origins from macrophages. Microglia are responsible for sensing and maintaining homeostasis in the CNS. Following an environmental stimulus, microglia adopt a series of different physical phenotypes ranging from pro-inflammatory activation to anti-inflammatory activation (Orihuela et al., 2016; Loane and Byrnes, 2010). Pro-inflammatory microglia produce NO, ROS, and a number of pro-inflammatory cytokines (Nakamura et al., 1999). Additionally, they release chemoattractants, such as monocyte chemoattractant protein (MCP-1), that draw more microglia to the site, increasing the inflammatory response. The anti-inflammatory phenotype produces anti-inflammatory cytokines and can be neuroprotective (Stein et al., 1992).

Microglia express IR as well as the IGF-1 receptor. They also express IRS-1 and IRS-2, which are required for propagation of insulin/IGF-1 signaling. To evaluate the anti-inflammatory effects of insulin, cultured human-glia derived human microglia were treated with varying doses of insulin, in the presence of a cocktail of known reactive factors, IL-6, TNFα, and IL-1β, for 48 h (Spielman et al., 2015). Low concentrations of insulin (10 pM and 1 nM), resulted in a pro-inflammatory phenotype, similar to astrocytes as discussed above, characterized by an upregulated media levels of IL-8 and MCP-1 from stimulated human microglia. At higher concentrations (100 nM), insulin reduced the media levels of both IL-8 and MCP-1. In our laboratory, a higher dose of insulin, 0.36 μM, was shown to have anti-inflammatory effects in the immortalized microglia cell line, BV2 (Brabazon et al., 2018). We found that after treatment with insulin for 24 h, LPS stimulated microglia showed significant reductions in NO, ROS, and TNF-α production, while simultaneously increasing phagocytosis. Additionally, BV2 cells treated with a known inducer of phagocytosis, TNF-α, showed a significant decrease in phagocytic activity when incubated with insulin for 1 h (Brabazon et al., 2018).

In an HIV/AIDS model of neurodegeneration, insulin reduced HIV replication in a dose dependent fashion in primary human microglia, with 3.0 IU/mL providing the greatest therapeutic efficacy (Mamik et al., 2016). In the same study, insulin significantly decreased the HIV-induced increase in pro-inflammatory genes IL-6, CXCL10, and IL-1β.

These studies establish the efficacy of insulin to ameliorate pro and promote anti-inflammatory cytokine release, improve phagocytosis, and halt deleterious viral replication, thus demonstrating insulin’s potential therapeutic value. However, additional research is needed to fully characterize microglial response to insulin.

### Macrophages

While not normally present within the CNS, macrophages are central mediators of neuroinflammation and contribute to inflammatory associated conditions, such as (Ieronymaki et al., 2019) neurotrauma and neurodegeneration (Hawkes and McLaurin, 2009). The effect of insulin on peripheral macrophages has been studied extensively, particularly in the context of obesity and diabetes (Olefsky and Glass, 2010). Individuals with insulin resistance and diabetes have high levels of the pro-inflammatory cytokines produced by macrophages, TNFα and IL-6 (Tajiri et al., 2005; Olefsky and Glass, 2010). Insulin treatment can significantly reduce iNOS expression and NO production in peripheral macrophages (Stevens et al., 1997).

Similar to neurons, chronic insulin exposure to macrophages can induce insulin resistance. Isolated primary thioglycolate-elicited peritoneal macrophages (TEPMs) from mice fed a high fat diet (HFD), consisting of 60% energy from fats for 7 days, have been used as a model of insulin resistance to study effects on inflammation. Isolated TEPMs, from mice on HFD, exposed to a 30 min insulin treatment (100 nM) displayed a decrease in AKT phosphorylation and significantly lower expression of IR compared to TEPMs isolated from mice fed normal diets. TEPMs exposed to high-dose (100 nM) insulin for 48 h showed reduced AKT phosphorylation and decreased IR expression following a 30 min re-stimulation of insulin (Ieronymaki et al., 2019). Furthermore, a downstream mediator of AKT, mTOR, is more active in insulin resistance macrophages, as demonstrated by significantly greater phosphorylation of mTOR in HFD TEPMs compared to isolated TEPMs from mice fed a normal chow diet for 7 days.

Treatment with insulin also prevented LPS induced pro-inflammatory responses in primary mouse peritoneal macrophage (Zhu et al., 2019). Cells stimulated with insulin (100 nM) and LPS (100 ng/mL) for 16 h, displayed significantly decreased mRNA expression of IL-6, IL-1β, NOS2, and COX2 and supernatant derived IL-6, IL-1 β, and TNF-α when compared to LPS treated cells. These results suggest an anti-inflammatory effect of insulin on pro-inflammatory activated macrophages.

With these cellular effects of insulin mind, we now move to consideration of insulin within different trauma and neurodegenerative conditions, to further explore the role of insulin in the CNS.

## Insulin in TBI

Traumatic brain injury is a major cause of death and disability in the United States. There are two primary injury types of TBI, focal and diffuse, although many injuries can present with both components. Focal injuries are direct area injuries resulting from collision or penetrative forces acting upon the skull and commonly present with contusions and subdural hematomas (Lifshitz, 2015). Diffuse injuries are most often caused by rapid acceleration then deceleration of the head (Mergenthaler et al., 2013) and can present with widespread tearing of axons and small vessels by shearing forces (Park et al., 2009; Andriessen et al., 2010). Concussions, often referred to as mild TBI, are considered a type of diffuse injury that commonly occur during sport activities and present with mild axonal damage (Signoretti et al., 2011).

Co-morbidities are not uncommon in TBI; head injuries greatly increase the risk of neurodegenerative diseases such as AD and chronic traumatic encephalopathy (Plassman et al., 2000). Additionally, patients with diabetes often experience exacerbated symptoms and are at higher risk of developing neurodegenerative diseases after TBI (Zimering et al., 2019).

Traumatic brain injury is a pathologically heterogenous disease, which can result in a variety of cognitive deficits depending on the location, type, and severity of damage (Andriessen et al., 2010). Up to 15% of individuals with a mild TBI report deficits with cognitive function a year after injury (Roe et al., 2009). These cognitive impairments can manifest as deficits in memory retrieval or deficits in task acquisition (Whiting and Hamm, 2008). Since learning and memory function are hippocampal dependent functions (Scoville and Milner, 1957; Lazarov and Hollands, 2016) these deficits can be attributed to the significant hippocampal atrophy often observed following TBI (Kotapka et al., 1992; Bigler et al., 1996; Vakil, 2005; McKee and Daneshvar, 2015).

Post TBI cognitive deficits can also result from cellular and metabolic dysfunction after injury, including inflammation, insulin resistance, and decreased cerebral glucose uptake. As a direct result of the injury, necrotic neuronal cell death can occur (Goodman et al., 1994; Colicos et al., 1996) and an increased number of microglia and astrocytes congregate in the area. Both activated microglia and reactive astrocytes produce excess cytokines which promote inflammation and can form scars that prevent axonal regeneration (Myer et al., 2006; Wang et al., 2018). Prolonged microglia activation after TBI correlates with neuronal cell death observed after injury (Chen et al., 2003).

These cellular alterations result in a fluctuation of glucose uptake and metabolism that have often been referred to as the neurometabolic cascade of concussion (Giza and Hovda, 2001), though this change is observed in all types of TBI. Acutely after injury, hyperglycemia accompanied by hyperglycolysis is observed as cells utilize ATP-requiring membrane ionic pumps in an effort to restore ionic and cellular homeostasis (Yoshino et al., 1991; Giza and Hovda, 2014). Following this initial burst of energy, the brain enters a hypometabolic state that can last for days to weeks after injury in pre-clinical models (Yoshino et al., 1991).

These alterations are observed in clinical work as well. Many patients demonstrate hyperglycemia, which has been found to be a predictor of poor neurological outcome (Young et al., 1989; Terzioglu et al., 2015). Additionally, diabetic patients, who can have both hyperglycemia and insulin resistance have a significantly higher risk of mortality after TBI (Ley et al., 2011). Regional cerebral hypometabolism has been observed in patients years after injury and is associated with cognitive and behavioral deficits (Gross et al., 1996; Byrnes et al., 2014). This period of cerebral hypometabolism is also a period of increased vulnerability to injury (Prins et al., 2013; Selwyn et al., 2016).

Hyperglycemia may be stress-induced, which is caused by activation of both the hypothalamic-pituitary-adrenal axis and sympathetic autonomic nervous system after TBI (Llompart-Pou et al., 2008). Both systems increase levels of glucagon, catecholamines, and cortisol, which drastically increase blood glucose levels (Peters et al., 2002). In addition, catecholamines stimulate islet beta cells to increase glucagon production, resulting in decreased insulin secretion (Halter et al., 1984). Reduced insulin sensitivity and signaling at synapses has been observed after TBI in animal studies, indicating that insulin resistance may occur and impair the body’s ability to maintain glucose homeostasis (Karelina et al., 2016; Franklin et al., 2019). The mechanism behind insulin resistance is not currently known, but may be related to post-injury excitotoxicity described in the previous section.

Our lab has shown that a “milder,” diffuse lateral fluid percussion injury in rats resulted in global cerebral hypometabolism that persisted from 3 h to 9 days post injury (Selwyn et al., 2013). We have also found that the [^18^F]-FDG uptake profile after moderate controlled cortical impact injury (CCI) is altered by injury and dependent on the cellular composition of the region of interest (Brabazon et al., 2016). These data show that CCI results in a significant hypermetabolic response in the hippocampus, which demonstrates both glial and neuronal effects of injury at 3 h post injury and returns to baseline by 1 to 10 days post injury. However, in the neuron-free region of the corpus callosum, CCI results in significant increases in glucose uptake at all time points examined, while the amygdala, which showed no glial change but marked loss of NeuN stain, demonstrated a significant decrease in glucose uptake from days 3 to 7 post injury. One can conclude from this that glial and neuronal glucose uptake is altered differently by injury, and this can change the observations made by ^18^fluorodeoxyglucose (FDG) positron emission tomography (PET).

### Insulin Therapy

Several clinical trials have aimed to treat hyperglycemia after TBI by glucose control via IV administration of insulin (Table 1), but results have been mixed, and insulin’s ability to penetrate into the CNS during these studies is rarely considered. One study found that maintaining low glucose levels through tight glycemic control had favorable outcomes, which led to intensive insulin therapy (IIT) being implemented in many intensive care units (van den Berghe et al., 2001). One study found that IIT improved neurological outcomes after 6 months and did not increase mortality rates in patients with severe TBI (Yang M. et al., 2009). However, multiple studies could not confirm these findings in TBI patients, and even found that IIT increased risk of mortality, cerebral metabolic crisis, and hypoglycemia (Bilotta et al., 2008; Oddo et al., 2008; Investigators et al., 2009; Vespa et al., 2012). A 2018 meta-analysis of tight insulin control found that maintenance of systemic glucose between 4.4 and 6.7 mmol/L using insulin led to a trend toward improved neurological improvement but increased the risk of developing hypoglycemia (Hermanides et al., 2018).

**TABLE 1:** Comparison of systemic versus intranasal delivery of insulin in treatment of TBI, SCI, AD and PD.

	**Systemic Insulin Administration**				**Intranasal Insulin Administration**			
	**Positive effects**	**Ref. #**	**Negative effects**	**Ref. #**	**Positive effects**	**Ref. #**	**Negative effects**	**Ref. #**
Traumatic Brain Injury	Improved neurological outcomes	van den Berghe et al., 2001; Oddo et al., 2008; Yang Y. G. et al., 2009)	Risk of hypoglycemia Increased mortality rate	van den Berghe et al., 2001; Bilotta et al., 2008; Yang Y. G. et al., 2009a; Vespa et al., 2012 Bilotta et al., 2008; Oddo et al., 2008; Investigators et al., 2009; Vespa et al., 2012; Hermanides et al., 2018	Increased glucose uptake Improved cognitive function Reduced inflammation	Brabazon et al., 2017 Brabazon et al., 2017 Beirami et al., 2017; Brabazon et al., 2017	N/A	
Spinal Cord Injury	Improved motor function in animal models Reduced apoptosis in spinal cord in animal models Reduced inflammation in spinal cord in animal models	Nagamizo et al., 2007; Wu et al., 2007; Yang Y. G. et al., 2009) Wu et al., 2007; Yang Y. G. et al., 2009) Wu et al., 2007	Hyperglycemia from exogenous administration causing pro-inflammatory state Insulin may not produce sufficient response Insulin resistance	Prakash and Matta, 2008; Torabi et al., 2018 Duckworth et al., 1980 Duckworth et al., 1980	N/A		N/A	
Alzheimer’s disease	Improved memory	Craft et al., 1996	Risk of hypoglycemia Insulin resistance	Craft et al., 1996 Zhong et al., 2012; Lutski et al., 2017	Reduced amyloid beta levels, repaired insulin signaling, alleviated cognitive deficits in animal models No hypoglycemia Improved cognition and working memory Improved delayed verbal and story recall task performances	Chen et al., 2014; Salameh et al., 2015; Mao et al., 2016; Guo et al., 2017 Hanson and Frey, 2008 Hanson and Frey, 2008; Reger et al., 2008; Claxton et al., 2015 Reger et al., 2006; Craft et al., 2012, 2017	Varied efficacy dependent on genotype	Craft et al., 2000, 2003
Parkinson’s disease	N/A		N/A		Alleviated motor deficits in animal model No impact on blood glucose levels and quickly detectable in CSF Increased verbal fluency Lowered disability scores Improved motor function	Pang et al., 2016; Fine et al., 2020 Pang et al., 2016 Novak et al., 2019 Novak et al., 2019 Novak et al., 2019	N/A	

Our previous work demonstrated that after a moderate TBI in rats, intranasal insulin administered within 4 h after injury significantly increased glucose uptake in the hippocampus, improved cognitive function, and reduced inflammation (Brabazon et al., 2017). In this study, insulin administered daily for 14 days significantly improved performance in a Morris water maze task as well as improved time to cross a beam during a beam walk task. This was accompanied by significant reductions in microglial number in the hippocampus, although significant changes in neuronal viability or lesion volume were not observed.

Intranasal insulin has been shown to be a promising method for decreasing microglial-induced inflammation in the CNS (Beirami et al., 2017; Brabazon et al., 2017). In addition to our work and other work *in vitro*, mice fed HFDs for 12 weeks demonstrated elevated levels of pro-inflammatory cytokine gene expression the hippocampus that were reduced by hippocampal infusion of insulin (2.64 μL of Humulin insulin per day at 4 mU/μL) (Gladding et al., 2018). With the decrease in inflammation, this therapy has also shown potential to decrease anxiety (Beirami et al., 2017), although research into TBI-induced psychiatric disorders is currently under-evaluated (Chapman et al., 2013; Brabazon et al., 2017). The literature on intranasal insulin is still limited, but additional research into the effects of insulin as a therapeutic approach after TBI are warranted.

## Insulin in SCI

Within the United States alone, there are approximately 12,000 new cases of SCI each year (NSCISC, 2016). This type of neurotrauma results in permanent motor, autonomic, and sensory function loss, related to the axon tracts injured. In addition to damage to white matter tracks, there is a loss of neurons in the gray matter, significant gliosis, including astrogliosis and formation of a gliotic scar, microgliosis, and infiltration of peripheral immune cells. All of these pathological events can have significant impact on glucose metabolic needs within the spinal cord.

After SCI, peripheral blood glucose is significantly altered. Female mice exposed to a thoracic contusion injury demonstrated elevations in blood glucose levels from 4 h through at least 2 weeks post-injury, accompanied by significant reductions in peripheral insulin levels (Jing et al., 2018). These changes in peripheral blood glucose may be related to alterations in perfusion and activity of pancreatic islet cells following SCI.

In mice exposed to SCI, insulin levels are reduced from 7 to 28 days post-injury (the longest time point studied) (Rouleau et al., 2007). Impairment of insulin signaling, such as by induction of a diabetes model via streptozotocin in rats, can significantly worsen the outcome of a SCI (Tariq et al., 1998). Motor function after a moderate compression thoracic injury was markedly worsened from 1 to 10 days after injury in streptozotocin treated rats. This impairment was accompanied by a reduction in glutathione levels in the diabetic animals, suggesting an impaired ability to respond to oxidative challenge.

Perhaps unsurprisingly, SCI is associated with an increase in occurrence of Type II diabetes and peripheral insulin resistance (Jeon et al., 2002). In a study of 45 SCI patients, 27% were found to have developed insulin resistance (Duckworth et al., 1980). A 2008 study in 42 SCI patients found that incidence of insulin resistance did not depend on injury severity or location, with equal levels amongst para- and tetraplegics and those with complete and incomplete injuries (Huang et al., 2008). Changes in muscle use and innervation have been suggested to be a primary influence on the development of insulin resistance (Graham et al., 2019), although aberrant activation of the sympathetic nervous system may also play a role (Karlsson, 1999). Increasing exercise can alleviate this condition, reducing blood glucose and increasing insulin sensitivity (Jeon et al., 2002; Koury et al., 2013). However, it is currently unclear if the central nervous system is also demonstrating insulin resistance after SCI, and this topic needs additional research.

Although CNS insulin resistance has not yet been evaluated after SCI, this type of injury has been found to significantly alter glucose uptake in injured spinal cord tissue; this may be due to changes in availability of peripheral glucose or alterations in ability of damaged tissue to take up glucose. Autoradiography examination of monkey spinal cord demonstrated that glucose metabolism was acutely increased for 1 h after injury (Anderson et al., 1980), followed by a depression that lasted at least eight additional hours (Rawe et al., 1981). These changes were observed in both white and gray matter at the lesion site (Schechter et al., 1990). In a rat model of moderate contusion SCI, we have found that injury resulted in a marked reduction in glucose uptake at 6 h post-injury, as measured by FDG-PET imaging (von Leden et al., 2016). This glucose uptake depression then returned to baseline levels and remained there throughout the following 2 weeks of the study, although other studies have suggested that the uptake of FDG increases after the first 24 h, to peak at 7 days post-injury (Nandoe Tewarie et al., 2010). Correlation with histological analysis showed a link between the depression and neuronal loss and inflammation.

In addition, we demonstrated that these changes in glucose uptake were dependent on subject age. Increasing the age of rats from 3 months (von Leden et al., 2016) to 12 months (von Leden et al., 2019) resulted in a significant alteration in the glucose uptake profile. Aged rats showed no significant change in glucose uptake acutely after injury, but did show a marked increase in glucose uptake by 14 days post-injury (von Leden et al., 2019). This increase was correlated with elevated inflammation in the lesion epicenter in the aged spinal cord.

Chronically, autoradiography studies have shown that glucose uptake and metabolism is depressed in the moderately injured rodent spinal cord (Horner and Stokes, 1995). Glucose utilization was found to be significantly depressed both above and below the level of the lesion through 3 months post-injury. Imaging of glucose uptake and metabolism with PET demonstrated a chronic decrease in FDG uptake in human chronic cervical myelopathy patients at 6 to 24 months after symptom onset (Floeth et al., 2010).

The effect of SCI on IR expression in the brain or spinal cord has not yet been evaluated. However, GLUT3 and 4, both of which show insulin dependence, have been shown to be elevated at both the protein and gene level 2 weeks after injury in both young and aged populations (von Leden et al., 2019). Previous work has shown that insulin can directly increase GLUT4 gene and protein expression (Valverde et al., 1999). Further, GLUT4 overexpression has been found to increase insulin sensitivity (Atkinson et al., 2013). While not studied directly, it is possible that elevated GLUT4 in the injured spinal cord may be either a response to increased insulin signaling, or a response intended to increase insulin signaling.

### Insulin Therapy

Studies have shown that increasing glucose uptake after SCI can have beneficial effects on recovery. Increasing GLUT4 expression by the small molecule FM19G11 led to accelerations in locomotor recovery after SCI in rats, accompanied by an increase in local neural progenitor cells (Rodriguez-Jimnez et al., 2012). However, caution is required, as hyperglycemia has also been associated with worsened outcome after SCI. Elevated blood glucose levels, produced by exogenous administration of glucose to microglia cells *in vitro* or injections of streptozotocin *in vivo* have been shown to increase pro-inflammatory markers and neuronal damage (Kobayakawa et al., 2014).

The use of insulin as a therapeutic is controversial (Table 1). Considering the peripheral insulin resistance after SCI discussed above, it is unclear if insulin would have sufficient receptor activation to produce a response. However, an early study showed that monocyte binding to insulin in SCI patients was normal, and not impaired by peripheral insulin resistance (Duckworth et al., 1980). This suggests that anti-inflammatory aspects of insulin activity may be unchanged by injury-induced insulin resistance. In addition, it is currently unclear if peripheral insulin resistance is replicated within the CNS, as it is in brain injury (Karelina et al., 2016). Future work is needed to determine if central insulin administration may be effective in ameliorating effects of SCI.

Intraperitoneal injection of insulin after a moderate-severe weight drop injury of the rodent spinal cord has been shown to significantly improve motor function. BBB scores of rats at 6–8 weeks after injury showed significant increases with insulin (3 IU/kg per day for 7 days, in combination with 3 g/kg of glucose per day) (Yang Y. G. et al., 2009). Combining this therapy with inhibition of chondroitin sulfate proteoglycans led to an additive effect. Treatment with insulin also demonstrated a small reduction in apoptotic cells in the injured spinal cord. Similarly, IP administration of insulin (1 IU/kg in combination with glucose) in a rat spinal cord compression model showed significantly improve motor scores, using the Tarlov and inclined plane tests (Wu et al., 2007). This was supported by electrophysiology showing increased evoked potential amplitude in hindlimbs and reduced spinal cord neuron apoptosis and inflammation with insulin treatment.

In a rabbit model of ischemic SCI, a single dose of insulin (0.5 IU/kg) peripherally 30 min prior to injury significantly improved motor function and neuronal survival at 7 days post-injury (Nagamizo et al., 2007). This peripheral insulin administration study also included a post-injury treatment group, which lowered peripheral glucose levels similarly to the pre-treatment group, but did not result in significant effects on motor recovery or neuroprotection. It is not clear if the benefits of the pre-insulin treatment were due to central or peripheral effects, and more investigation is needed into the role of pre-injury glycemic status on outcome after SCI. However, it has been shown that hyperglycemia can contribute to a pro-inflammatory state that may impair outcome (Prakash and Matta, 2008; Torabi et al., 2018).

These data demonstrate that insulin delivery after SCI has potential, although a risk of hypoglycemia suggests treatment should proceed carefully. As in TBI, central administration of insulin may prove to be a useful alternative. However, essential basic biology into IR expression and effects of SCI are needed to further explore therapeutic options.

## Insulin in Neurodegenerative Disease

**FIGURE 2:**
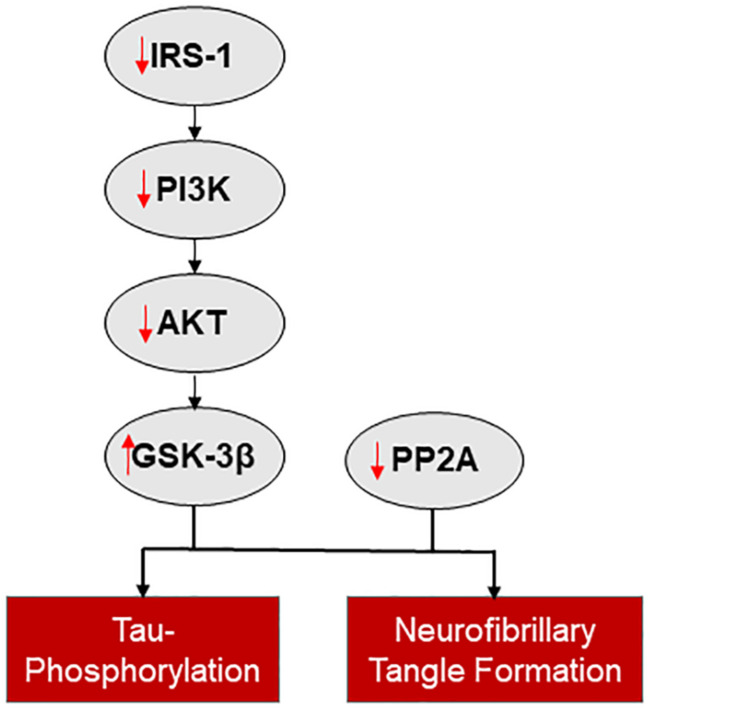
The connection between insulin signaling and neurodegenerative disease. Reduced insulin signaling is associated with increased tau-phosphorylation and neurofibrillary tangle formation, via a reduction in IRS-1 mediated activation of the PI3K/AKT pathway and disinhibition of GSK-3β and reduced PP2A.

Insulin signaling impairment and insulin resistance have been observed in neurodegenerative diseases such as AD (Talbot et al., 2012) and PD (Hogg et al., 2018) (Figure 2). Diabetes mellitus, with a reduction in insulin sensitivity or insulin release, is a comorbidity of both AD and PD, with an increased risk of developing these neurodegenerative disorders in patient with diabetes (Arvanitakis et al., 2004; Hu et al., 2007; Yang et al., 2017). Insulin resistance leads to decreased uptake of insulin and a reduced sensitivity of IRs in the brain (Messier and Teutenberg, 2005) and is linked to cognitive decline in diabetes, AD, and PD (Brands et al., 2005; Messier and Teutenberg, 2005; Bosco et al., 2012).

### Alzheimer’s Disease

Alzheimer’s Disease is the most common neurodegenerative disease and form of dementia. It is characterized by cognitive and functional decline over time (Huang and Mucke, 2012). Amyloid plaques and neurofibrillary tangles are two major pathological markers of AD in the brain (Braskie et al., 2010). Glucose uptake is markedly decreased in patients with AD indicating impaired glucose metabolism (Mosconi, 2013; Calsolaro and Edison, 2016).

Studies have shown that AD presents with its own neurometabolic response prompting some researchers to refer to AD as “type 3 diabetes” (de la Monte, 2014). This term refers to the cerebral insulin resistance and reduction in glucose metabolism observed in AD. These data have lent themselves to a more in-depth examination of the effect of insulin on cell populations of the CNS beyond the GLUT response.

A major branch of the insulin signaling pathway, the PI3K/AKT pathway, is downregulated in AD and this change may be a major contributor to insulin resistance (Gabbouj et al., 2019). IRS1 is a homeostatic regulator of PI3K signaling (White, 1998; Metz and McGarry Houghton, 2011). Abnormal serine phosphorylation of IRS1, which, when co-localized with neurofibrillary tangles, hinders the actions of insulin (Yarchoan et al., 2014) and is associated with cognitive decline (Talbot et al., 2012). When the PI3K/AKT pathway is downregulated the expression of two downstream targets, GSK-3β and protein phosphatase 2A (PP2A), is altered (Ghasemi et al., 2013). GSK-3β expression is disinhibited and PP2A expression is inhibited with loss of PI3K/AKT activity; both changes increase tau-phosphorylation and neurofibrillary tangle formation (Planel et al., 2007; Avila et al., 2010). Additional pathways potentially involved in insulin resistance in AD are reviewed by Ferreira et al. (2018).

### Parkinson’s Disease

Parkinson’s disease is the second most common neurodegenerative disorder and is characterized by decreased movement, walking instability, tremors, and associated dementia (Jankovic, 2008). Many of the symptoms observed are due to a loss of dopaminergic neurons and the production of Lewy bodies and neurofibrillary tangles in the brain (Bernheimer et al., 1973; Braak and Braak, 2000; Compta et al., 2014).

Insulin plays a role in dopamine release in the brain; when present insulin enhances dopamine uptake via the PI3K pathway (Carvelli et al., 2002) and dopamine release via increased excitability of cholinergic interneurons which activate nicotinic acetylcholine receptors (Stouffer et al., 2015). Similarly to AD, the PI3K/AKT pathway is altered in PD, specifically with an overexpression of GSK-3β, leading to increased neurofibrillary tangle formation contributing to PD dementia (Yang et al., 2018).

### Insulin Therapy

Intranasal insulin has been explored as a treatment for AD and PD in both animal and clinical studies (Table 1). It has been shown in multiple AD mouse models (the 3xTg mouse model, App/PS1 mouse model, and SAMP8 mouse model) and in the Streptozotocin rat model, a diabetes model that develops AD-like symptoms, that both acute (1 to 2 weeks) and chronic (6 weeks) intranasal insulin treatment reduces amyloid-B levels and repairs insulin signaling through downregulation of tau kinases such as GSK-3β, and alleviates cognitive deficits associated with the models (Chen et al., 2014; Salameh et al., 2015; Mao et al., 2016; Guo et al., 2017).

In the six OHDA rat model of PD, both a daily high dose (12IU) for 2 weeks and a daily low dose (3IU) for 4 weeks of intranasal insulin alleviated motor deficits observed in the model in overall locomotor activity and in a variety of motor behavioral tests (Pang et al., 2016; Fine et al., 2020). Pang et al. (2016) demonstrated that insulin delivered intranasally did not affect body weight or blood glucose levels, but was detectable in the CSF within minutes of administration. In addition, insulin increased the number of surviving tyrosine-hydroxylase positive neurons by 75%.

In 2008, Dr. William Frey II’s group at the University of Minnesota reported that intranasal insulin was effective in improving memory for patients suffering from AD disease, and the patients suffered no adverse effects of decreased blood sugar (Hanson and Frey, 2008). Clinical research of AD has demonstrated that intranasal insulin treatment improves cognition and memory performance. A three-week daily administration of insulin improved delayed story recall and increased functional status (Reger et al., 2008). Additionally, insulin administered over 4 months daily increased both delayed verbal and story recall tasks (Craft et al., 2012, 2017). Evaluation of the mechanism by which insulin improves outcomes in AD has demonstrated a multifaceted therapy; insulin can directly affect neurons by increasing glucose uptake or improving viability via non-metabolic pathways, while also contributing to the direct degradation of beta amyloid via insulin-degrading enzyme activation, downregulation of GSK-3β, and reducing cortisol expression, which can inhibit hippocampal glucose uptake (for review, see Frey, 2013).

Additionally, in studies comparing memory impaired/AD participants with and without a risk factor for late onset AD, the ApoE e4 allele, insulin effects varied based on the genotype (Craft et al., 2000, 2003). Intranasal administration of normal insulin improved verbal memory in subjects without the allele in story recall and word list learning tasks (Reger et al., 2006), however intranasal administration of insulin detemir, a long-acting form of insulin, improved verbal memory in adults who were carriers for the allele and improved working memory for all participants (Claxton et al., 2015). This indicates that genotype plays a role in severity of AD symptoms and may require a longer acting treatment to have a similar effect.

In a recent PD clinical trial, intranasal insulin treatment caused increased verbal fluency (FAS) in participants that received treatment, while the placebo group had decreased FAS scores indicating that there was improvement in cognition in the treated group. Participants treated with insulin also had lower disability scores compared to the placebo group, demonstrating improvement in motor function and overall performance with treatment (Novak et al., 2019).

Intranasal insulin has been shown to produce beneficial effects in healthy participants. It can enhance feelings of self-confidence and well-being, thus improving overall mood, as well as aid in word recollection in a word list learning task (Benedict et al., 2007). Improvements to memory are gender dependent. Women demonstrated improved hippocampus-dependent memory and working memory with intranasal insulin, while men did not (Benedict et al., 2008).

Another method of insulin delivery, IV administration, in AD patients has showed improved memory performance (Craft et al., 1996), but long-term treatment poses a risk of hypoglycemia. Insulin resistance has also been suggested to lead to decreased levels of insulin crossing the BBB (Zhong et al., 2012; Lutski et al., 2017). Intranasal insulin avoids the risk of hypoglycemia and bypasses insulin resistance due to direct delivery to the CNS, thus is a more sustainable long term treatment. More clinical trials need to be conducted to further explore the benefits of intranasal insulin. The current studies have been relatively small in sample size, and have only given treatment over 4 months, which is nothing compared to the duration of the diseases. Collectively, the information above indicates that intranasal insulin is a promising treatment for both AD and PD.

## Conclusion

The therapeutic value of insulin and the deleterious effect of insulin resistance on neuronal and glia cells, as it related to neurotrauma and neurodegeneration, has only recently been explored. *In vitro* and *in vivo* studies demonstrate insulin plays a significant role in cellular metabolism in all brain cells and disruption of insulin signaling, from insulin resistance, can contribute to dysfunction and pathological conditions as observed in AD and PD. Clinical trials using insulin, particularly intranasal insulin, show promise in patients with AD and PD, as treatment leads to restoration of insulin signaling and amelioration of cognitive and motor deficits. Our lab has shown the therapeutic effects of intranasal insulin in an experimental model of TBI, that improves memory, increase glucose uptake, and decreases neuroinflammation and hippocampal lesion volume. This review highlights the therapeutic value of insulin in the context of neurotrauma and several neurodegenerative disorder and describes the cellular effects of insulin resistance on multiple brain cells. Further research is warranted to identify dosing and schedule of insulin treatment to improve outcomes for patients suffering from neurological disease and trauma, and to understand the role insulin resistance plays in the development and progression of brain disorders.

## Author Contributions

KB designed the overview of the manuscript and wrote spinal cord injury section. MS researched and wrote *in vitro* section. DA researched, wrote neurodegeneration section, and designed figures. FB, DA, and NH researched and wrote traumatic brain injury section. FB and MS wrote introduction. All authors contributed to the article and approved the submitted version.

## Conflict of Interest

The authors declare that the research was conducted in the absence of any commercial or financial relationships that could be construed as a potential conflict of interest.
